# Reconsidering the role of patriarchy in upholding female genital modifications: analysis of contemporary and pre-industrial societies

**DOI:** 10.1038/s41443-022-00581-5

**Published:** 2022-06-14

**Authors:** Ellen Gruenbaum, Brian D. Earp, Richard A. Shweder

**Affiliations:** 1 Department of Anthropology, Purdue University, West Lafayette, IN USA; 2 Uehiro Centre for Practical Ethics, University of Oxford, Oxford, UK; 3 Department of Comparative Human Development, University of Chicago, Chicago, IL USA

**Keywords:** Translational research, Risk factors

## Abstract

According to the World Health Organization (WHO), customary female genital modification practices common in parts of Africa, South and Southeast Asia, and the Middle East are inherently patriarchal: they reflect deep-rooted inequality between the sexes characterized by male dominance and constitute an extreme form of discrimination against women. However, scholars have noted that while many societies have genital modification rites only for boys, with no equivalent rite for girls, the inverse does not hold. Rather, almost all societies that practice ritual female genital modification also practice ritual male genital modification, often for comparable reasons on children of similar ages, with the female rites led by women and the male rites led by men. In contrast, then, to the situation for boys in various cultures, girls are not singled out for genital modification on account of their sex or gender; nor do the social meanings of the female rites necessarily reflect a lower status. In some cases, the women’s rite serves to promote female within-sex bonding and network building—as the men’s rite typically does for males—thereby counterbalancing gendered asymmetries in political power and weakening male dominance in certain spheres. In such cases, and to that extent, the female rites can be described as counter-patriarchal. Selective efforts to discourage female genital modifications may thus inadvertently undermine women-centered communal networks while leaving male bonding rites intact. Scholars and activists should not rely on misleading generalizations from the WHO about the relationship between genital cutting and the social positioning of women as compared to men. To illustrate the complexity of this relationship, we compare patterns of practice across contemporary societies while also highlighting anthropological data regarding pre-industrial societies. Regarding the latter, we find no association between the presence of a female initiation rite and a key aspect of patriarchy as it is classically understood, namely, social endorsement of a gendered double-standard regarding premarital sexual activity. We situate this finding within the broader literature and discuss potential implications.

## Introduction

Diverse challenges are faced by immigrants who move from Global South settings where both female and male genital modifications^1^ are customary, viewed as symbolically linked, and widely regarded as desirable or virtuous, to regions of the Global North where immigrant-associated female modifications of all types are strongly stigmatized [2], while the corresponding male modifications within the same communities are either ignored, tolerated, or viewed positively [3–6]. A common explanation for these discrepant attitudes is that the female modifications are markedly more harmful than the male modifications, with more severe adverse consequences for health and sexuality. It is also widely believed that the female modifications, but not the male modifications, reinforce male dominance and female subordination within the practicing cultures, in violation of the principle of gender equality.

Despite the popularity of these views among activists and policymakers, they have been challenged by scholars working within various traditions including African studies and postcolonial feminism. These scholars claim that such binary thinking about female versus male genital modification is inaccurate. In particular, it reflects the widespread use of extreme, non-representative cases to illustrate the female practices in media reports and policy materials, while simultaneously depicting and discussing only the milder of the male practices, such as medicalized penile circumcision in the United States [7–11]. This results in the inapt conflation of multiple distinct versions of both kinds of modification. It also leads to stereotyping and homogenization of the dozens of culturally diverse communities that practice female and male genital modification together (along with their multifarious reasons for doing so) [12–15]. Finally, it reflects overly simplistic theories about gender, power, and social status in “non-Western” societies [16–21].

These criticisms are not without basis. In terms of physical implications, for instance, the level of harm or benefit caused by genital cutting is a function of several factors that do not reliably track biological sex categories. For example, it depends on the subtypes of cutting that have been adopted within a group and the extent to which each has been medicalized (that is, performed by a healthcare provider using sterile instruments, as is increasingly common for both male and female procedures in many contexts) [22–24]. Both types of cutting vary across cultures, with commensurate functional-anatomical effects in many cases [25–32]. Procedures range from those affecting the genital prepuce only (either the penile foreskin or the clitoral hood) [33–35], to ceremonial pricking of the clitoral or penile glans (as in some forms of female ‘circumcision’ or *hatafat dam brit* for circumcised male converts to Judaism) [36–38], inserting sharp objects into the penile urethra to induce bleeding (a traditional practice for boys in Papua New Guinea) [39], excision of labial tissues without clitoral modification (similar in scope to so-called ‘cosmetic’ labiaplasty) [40], ritual slitting of the penis including urethrotomy (subincision) [41–43], and partial or total removal of the protruding tip (i.e., most distal portion) of the clitoris, with or without infibulation [44–46].

The underlying motivations for these practices vary from culture to culture but are often broadly similar for both sexes, as we will discuss [47–51]. In some cultures, the rite for girls elevates their social status, promoting within-sex bonding and increased personal and political agency [52–54]. In others, where only boys are cut, it is the *lack* of a similar rite for girls that reflects their lower status [55–58]. No single, overarching rule can be applied to every context. As one anthropologist has stated: “By collapsing all of the many different types of procedures performed into a single set for each sex, categories are created that do not accurately describe any situation that actually occurs anywhere in the world” [59] (p. 3).

Ultimately, the stigma surrounding, and negative reactions to, the female-affecting practices arise for many reasons, including a genuine concern for the well-being of women and girls. However, Western-led campaigns to criminalize and eliminate only the female half of traditionally gender-inclusive genital cutting rites, mainly in Africa, have attracted growing criticism, including from feminist scholars who share female-child-protective concerns [15]. According to some critics, such sex-selective campaigning may work against gender equality—for example, by weakening women’s authority relative to men—while evincing moral double standards, the inconsistent application of human rights principles, and Western cultural imperialism [15, 16, 26, 48, 50, 60–65].

These concerns are not merely academic. Rather, a failure to understand the complexities of, and commonalties between, male and female genital cutting practices has real-world consequences. It can lead to harmful or inappropriate medical care for affected women, based on inaccurate assumptions about their bodies and experiences [66–68]; racially discriminatory surveillance of young girls and their families following migration to Western countries [69]; unjust criminal proceedings targeting certain minority groups [70, 71], and unequal treatment of persons of different sexes, genders, ethnic backgrounds, or religions [72–77]. Accordingly, some scholars and advocates have called for greater accuracy in public health campaigns and social initiatives regarding these practices, irrespective of one’s moral attitudes toward them [51, 78–81]. In other words, given the significant harms to already-marginalized individuals and communities that are caused by misinformed policy measures in this area, efforts to discourage female genital modifications, if they are to be justified, must at least be based on reliable information about the variegated nature of these practices rather than unsupported generalizations [82–84].

Here, we focus on one such generalization that has featured heavily in Western law and policymaking. As alluded to previously, the usual understanding in migrant-receiving countries is that “female genital mutilation” or “FGM”— as the relevant cluster of practices is more commonly known in such countries—is an expression of patriarchal power: the cutting is seen as something that men, whether directly or indirectly, impose on women and girls primarily to limit their freedom and “control their sexuality”. As such, “FGM” is thought to be evidence of, or to constitute, misogynistic oppression of women and girls. Alternatively, if it is acknowledged that women themselves carry out the ritual in most cases, express that they find value in it, and often strongly support it, this may be reflexively dismissed as internalized misogyny or false consciousness [76, 77, 79, 85–87]. Either way, the women are cast as “prisoners” of a custom whose underlying cultural logic or primary social function is their own subordination.

The World Health Organization (WHO) is often cited as a source of authority for this perspective, which we term the *patriarchy hypothesis*. Commonly, reference is made to the WHO’s assertion that “FGM” is an “extreme form of discrimination” against women and girls reflecting “deep-rooted inequality between the sexes” (implying male dominance) [88] (p. 1). As noted, however, numerous interdisciplinary studies over the years have put pressure on the patriarchy hypothesis, especially when considered as a unifying or comprehensive explanation of customary female genital cutting. This has prompted a re-examination of common background assumptions and evidence. The aim of this paper is to briefly summarize, critically evaluate, and then expand on the recent literature that has taken up this re-examination.

As a part of this, we present an empirical evaluation of anthropological data regarding pre-industrial societies, looking into the relationship between the presence of a female initiation rite in any given society and whether that society endorses a gendered double-standard regarding premarital sexual activity. We also discuss contemporary patterns of practice and potential cross-cultural links between gender (in)equality and the prevalence of female and male genital modification rites.

### Varieties of female genital modification

The term “FGM” has been adopted by the World Health Organization (WHO) to describe all medically unnecessary cutting of the external female genitalia. In principle, this includes, or should include, so-called “cosmetic” genital surgeries such as labiaplasty, clitoral unhooding, and vaginal “rejuvenation”, as the WHO does not distinguish between medicalized and non-medicalized forms of cutting, nor between (typically) voluntary and involuntary cutting in its definition [89–91]. These lacking distinctions may seem puzzling, given the centrality of concerns about health risks as well as notions of autonomy and informed consent to longstanding debates about the permissibility of different forms of genital cutting. However, we will not be exploring such moral debates in this paper.^2^ In practice, the WHO and other activist organizations focus exclusively on non-Western-associated forms of female genital cutting, such as those that are more prevalent in parts of Africa, South and Southeast Asia, and the Middle East, regardless of whether the cutting is done in a medicalized manner or whether woman or girl consents.

Globally, about 90% of customary female genital modifications range from (1) procedures affecting the clitoral prepuce or hood that are typically less substantial than male circumcision as performed within the same communities (these are the most common forms of female and male genital cutting, respectively, in much of Southeast Asia, for example), to (2) cutting or excising tissue from the labia with or without removing part or all of the externally visible portion of the clitoris (glans clitoris) along with tissues from the prepuce (WHO FGM Types 1, 2, or 4) [51].

The least common type of ritual female genital modification is infibulation (WHO FGM Type 3), which refers to the narrowing of the vaginal opening by cutting and repositioning the labia to form a covering seal. This form, which is most strongly associated with efforts to ensure female sexual abstinence before marriage, makes up about 10% of customary female genital modifications globally and is geographically concentrated in parts of Northeast Africa. It is also disproportionately used to illustrate the concept of “FGM” in Western media [17, 21, 51].

### Genital cutting as sex discrimination

Having clarified the range of practices classified as mutilations by the WHO, we can turn our attention to the set of claims implied by the patriarchy hypothesis. In a previous issue of this journal, the medical anthropologist Sara Johnsdotter and one of the present authors (Earp) summarized recent scholarly critiques of these claims, including the idea that all customary female genital modifications, especially those associated with Africa, must be rooted “in male dominance, sexist discrimination, or a controlling desire to undermine female sexual enjoyment”[1] (p. 201). Among numerous other critiques of this notion, Earp and Johnsdotter drew on a 2012 consensus statement by a group of anthropologists, social scientists, medical researchers, legal scholars, geographical area specialists, and feminists with longstanding expertise in the subject [51]. Two of us (Gruenbaum and Shweder) contributed this statement.

With our colleagues, we argued that the relationship between gender and genital cutting is not straightforward, whether in Africa or elsewhere [51]. First, we stressed that, almost without exception, girls are nowhere “singled out” for genital cutting on account of their gender (whereas boys in many cultures are) [80]. Instead, almost all societies that practice ritual female genital cutting also practice ritual male genital cutting, with variable physical and emotional implications that overlap between the sexes. Where both rituals occur together, the ceremonies are often viewed as equivalent by the practicing community and are referred to with the same local word [15, 50, 51, 80].

The rituals often also serve similar social functions, as when they form a part of an adolescent rite of passage into adulthood. Such rites often require both boys and girls, at a similar stage of development, to show courage in response to a painful trial, demonstrating their maturity and readiness for marriage and other adult privileges [15, 49–51, 95]. In such contexts, neither practice is typically intended to undermine the initiate’s ability to enjoy future sexual interactions. As Lori Leonard notes, the cutting of genitals in many cases is “construed as having little to do with sex, per se. Rather, its function is to prepare young men and women to occupy a preordained social role within the community” [18] (p. 162).

These “preordained roles” are, of course, typically gendered, with boys aspiring to become “real men” and girls aspiring to become “real women” in accordance with context-relative norms for masculinity and femininity [96]. These norms, which often evolve over time in response to changing social and material conditions, are not uniform across African societies. According to Michela Fusaschi, anthropologists have long viewed male and female genital cutting “as symbolically linked, complementary practices within a sex/gender system… *both* practices [working] together to support, reinforce, and reproduce gendered relations” in line with extant cultural ideals [95] (p. 2). Importantly, however: “these gender roles or norms were not necessarily organized around a hierarchical principle of female oppression and subordination; rather, prescriptive norms for men and women have varied widely across practising cultures, with power or status hierarchies often operating over age, for example, more than sex or gender per se” (*ibid*).

Nevertheless, where prescribed roles for men and women are asymmetrical in power and status within certain domains, the socialization that accompanies the genital cutting rites will tend to reflect and reinforce these asymmetries. Yet that is true of gendered socialization processes in all cultures with such asymmetrical roles, irrespective of genital cutting. Indeed, as we will discuss, some societies with the most highly asymmetrical gender roles, characterized by male dominance across multiple domains, do not practice any form of female genital cutting, but rather only cut the genitals of boys. At the same time, some societies that practice female cutting (and thus also male cutting) have gendered roles that are not traditionally construed as, or locally understood to be, asymmetrical, but rather as complementary and to be valued equally [50, 77, 97].

With respect to claims of “sexual control”, we note that efforts to temper or manage the sexual passions—often associated with adolescent immaturity—are widely thought to be required by responsible parenting and moral self-development in many cultures, including “Western” ones. In England and the United States, for example, both male and female genital operations, including penile circumcision, breaking of clitoral adhesions, and removal of the clitoral hood, were used for decades to combat childhood masturbation [98–102]. Genital cutting for this purpose was recommended by certain segments of the medical profession until the 1950s. Although female genital cutting of minors has since fallen out of favor in these countries, male genital cutting of minors persists. And while penile circumcision is no longer commonly advocated for reasons of “sexual control”—at least outside of highly conservative religious communities—British and American parents continue to employ all manner of techniques, including shame-based psychological pressures, to discourage “excessive” or “inappropriate” sexual expression in their adolescent children. Whatever one thinks of the wisdom of such parenting, it is not peculiar to African cultures, nor only directed at girls, whether or not genital cutting is involved. Indeed, in some African cultures that modify children's genitals, male initiates are taught that “promiscuity” is something that “boys” engage in, not respectable “men” [103]. In others, a “quieter” sexual organ, believed to result from cutting of the penis, is thought to help the boy focus his attention less on bodily temptations and more on his work, or on spiritual matters [61, 98, 100]. Contrastingly, some cultures that practice female genital cutting—and thus also male genital cutting—are relatively sexually liberal and, as such, are not particularly concerned with female chastity or virginity [17, 54, 104].

Given such variability, it cannot be maintained that female genital cutting as such (much less only female genital cutting) serves primarily to impair sexuality or otherwise subordinate the initiate on grounds of gender. Indeed, as we will now suggest, in at least some communities, the female rites in practice serve counter-patriarchal functions. This appears to be the case, for example, when the women-led ceremonies act as sources of female empowerment, solidarity, and network-building, operating as sites of resistance to male dominance in the public sphere [52, 54, 64, 104–106]. Examples of such solidarity can be found in the Bondo women’s society initiation rituals of West Africa, which includes spiritual challenges, learning women’s skills and secrets, being recognized as a woman, and coming under the protection of the society, as well as undergoing genital modification [17].

A similar description applies to female initiations among the Kuria and the Kalenjin of Kenya, for instance, among many other Central and East African cultures. In these cultures, bonds of attachment are strengthened among the female initiates, high-status women leaders, and other kinswomen. Enacting the traditional “circumcision” ritual has been a central, intense, and deliberately structured and shared physical and emotional experience, serving as a basis of mutual recognition, aid, and support throughout life [50]. Among the Hadza of Tanzania, the women’s bonding ritual, including genital cutting, has been argued to be a vital social and institutional factor counterbalancing male ritual power, thus enabling and ensuring gender equality in this “famously egalitarian” group [53].

Accordingly, as we and our colleagues have argued elsewhere, campaigns to eliminate female-only genital modifications can actually “weaken female power centers within society and bring women’s bodies and lives under the hegemonic control and management of local male religious and political leaders” [51] (p. 23). Consistent with this perspective, where “FGM”—that is, the ritual managed by women—has been criminalized in African countries, it has largely been in response to Western pressure on local male elites, who may be all too happy to use their legal and political power to undermine the women-centric initiations while their own, male-centric initiations are allowed to continue undisturbed [96]. It is not surprising, then, that in many contexts, it is the women themselves who are most strongly resistant to seeing their (and only their) customs made illegal [77, 107–111].

Consequently, rather than abandoning female genital modifications in response to such pressures, the women often take the practice underground, where it may lose many of its ceremonial elements [112–114]. They may also perform the procedure on younger and younger girls, even infants, who may be less likely to remember or report on the practice, but who are also less able to appreciate its cultural meanings [48, 106, 114, 115].

### Gender-asymmetrical sexual norms and genital modification

To summarize what has been said so far, female genital cutting serves multiple different functions in different societies—and often within the same society—that vary depending on the circumstances, including the details of the gender system within each group. Some of these systems are not primarily oriented around the subordination of women, nor does the female rite invariably perform such a role. In other cultures, by contrast, the cutting of girls and associated explicit or implicit socialization may indeed reflect—and serve to reinforce—asymmetrical gender norms, including a stricter concern for female chastity or virginity. Whether that is the case, however, is a function of the underlying cultural norms, not the fact of genital cutting (per se).

In many contemporary human societies, as we noted in the previous section, it is considered virtuous to practice restraint or moderation in sexual activity. Although typically applicable to both sexes, this perceived virtue is often gender-asymmetrical: that is, women and girls, compared to men and boys, are in many cultures more strongly expected to control their sexual impulses at least before marriage; they are also usually more severely punished for being perceived to violate these expectations, as can be seen, for example, in the practice of so-called honor killings [116]. This asymmetry is often interpreted as a sign of patriarchy, where patriarchy is broadly understood to refer to a social system in which men as a class have more power and authority across multiple domains than do women as a class and in which men maintain their superior status through structural domination and oppression of women [117].^3^

How does this picture relate to genital modification? A very misleading way to answer this question would be to focus on the subset of societies that have the most highly patriarchal gender roles or sexual norms and which also practice female (and male) genital modifications. One might then make note of the more or less explicit cultural associations within these societies between the female genital modifications and gender-asymmetric expectations of sexual restraint [119]. Finally, one could extrapolate from this non-representative situation to all societies in which female (and male) genital modifications are practiced, based upon a generalized assumption of cultural homogeneity among practicing groups. Something like this approach appears to have been taken by the WHO.

But such an approach leads to distorted theorizing [17]. A less biased approach would be to start with a larger sample of cultures that are variable in their gendered sexual norms, allowing for the possibility of cultural heterogeneity among practicing groups and a diversity of social functions and symbolic meanings. It can then be asked whether there is a robust relationship between the extent of gender inequality in a society—including with respect to sexual double standards—and the existence or prevalence within that society of female genital modification. To address this question, we will, in the following section, summarize a study by Martin Whyte of pre-industrial societies and describe a small preliminary analysis of two relevant variables in his data set: the presence of female initiation ceremonies (including those with genital modifications) and gendered premarital sexual norms. We find no consistent relationship between these variables. With respect to contemporary societies, the question has not been systematically studied, but several factors will bear on the answer:

1. First, of the many contemporary societies that can plausibly be described as patriarchal in the above sense, most do not practice customary female genital modifications. Rather, the most common situation among patriarchal societies, including those with the most highly asymmetrical gender norms and/or pervasive practices of female sexual control (e.g., Afghanistan), is that they either do not customarily modify the genitals of either sex, or they modify the genitals of males only [51].
2. Second, as noted, virtually every society that does practice customary female genital modification also practices customary male genital modification, often as a rite of passage into adulthood. Girls are not targeted for cutting. This is shown in Table 1. The table shows the number and percentage of countries around the world (*n* = 237 countries) with high versus low prevalence rates of male and female genital modifications. Although the ideal unit of analysis for a comparison of this sort is at the level of cultural communities or cultural regions rather than nation-states, that type of prevalence data is not readily available. Nevertheless, the country-level results in Table 1 are revealing. Of the 237 countries where prevalence data are available there are none (zero) where only female genital modifications are prevalent. Rather, wherever female genital modifications are prevalent so too are male genital modifications prevalent. Of the included countries, 60% have low prevalence for both male and female genital modifications; 30% feature high levels of male genital modifications and low levels of female genital modifications; and 10% of the countries feature high levels of both. We invite further analyses of this sort using different measures and units of analysis.

**Table 1:** Number (and global percent) of countries with high vs low prevalence of male and female genital modifications across 237 countries.

Genital Modifications (Prevalence)		Female**	
		High	Low
Male*	High	24 (10%)	70 (30%)
	Low	0	143 (60%)

*Male data source: [138]. Male prevalence estimations of “High” versus “Low” are based on country-specific prevalence rates for males 15 and older in 237 countries, compared to the calculated global prevalence estimation (37.7%) of all circumcised men in the target age group (15 and older) divided by all men in the target age group. Countries with prevalence rates above 37.7% were counted as “High” and those below were counted as “Low”.

**Female data source: [139]. Percentage of girls and women aged 15–49 years who have undergone FGM (2021). . Female prevalence globally was calculated differently, but still reflects relative high/low rating. Using country-specific prevalence rates in UNICEF data for females aged 15–49 for the same 237 countries, we averaged the prevalence rates without weighting for size of country, which yielded an average rate of 5.59%. All countries with rates above 5.59% were counted as “High” and those below were counted as “Low”.

Category I (male high/female high): Benin, Burkina Faso, Central African Republic, Chad, Cote d’Ivoire, Djibouti, Egypt, Eritrea, Ethiopia, Gambia, Guinea, Guinea-Bissau, Kenya, Liberia, Maldives, Mali, Mauritania, Nigeria, Senegal, Sierra Leone, Somalia, Sudan, Tanzania, Yemen (24 countries in total).

Category II (male low/female high): None.

Category III (male high/female low): Afghanistan, Albania, Algeria, American Samoa, Angola, Azerbaijan, Bahrain, Bangladesh, Bosnia & Herzegovina, Brunei, Burundi, Cameroon, Cocos (Keeling), Comoros, Congo, Democrat Republic Congo, Republic Cook Islands, Equatorial Guinea, Fiji, French Polynesia, Gabon, Gaza Strip, Ghana, Guam, Indonesia, Iran, Iraq, Israel, Jordan, Kazakhstan, South Korea, Kosovo, Islands, Kuwait, Kyrgyzstan, Lebanon, Lesotho, Libya, Madagascar, Malaysia, Morocco, Mozambique, Nauru, New Caledonia, Niger, Niue, Northern Mariana Is, Oman, Pakistan, Palau, Philippines, Qatar, Samoa, Saudi Arabia, Solomon Islands, South Africa, Syria, Tajikistan, Togo, Tokelau, Tonga, Tunisia, Turkey, Turkmenistan, Tuvalu, United Arab Emirates, United States, Uzbekistan, Vanuatu, West Bank, Western Sahara (70 countries in total).

Category IV (male low/female low): Andorra, Anguilla, Antigua & Barbuda, Argentina, Armenia, Aruba, Australia, Austria, Bahamas, Barbados, Belarus, Belgium, Belize, Bermuda, Bhutan, Bolivia, Botswana, Brazil, British Virgin Islands, Bulgaria, Burma, Cabo Verde, Cambodia, Canada, Cayman Islands, Chile, China, Christmas Island, Columbia, Costa Rica, Croatia, Cuba, Curacao, Cyprus, Czech Republic, Denmark, Dominica, Dominican Republic, Ecuador, El Salvador, Estonia, Falkland Islands, Faroe Islands, Finland, France, Georgia, Germany, Gibraltar, Greece, Greenland, Grenada, Guatemala, Guernsey, Guyana, Haiti, Holy See (Vatican), Honduras, Hong Kong, Hungary, Iceland, India, Ireland, Isle of Man, Italy, Jamaica, Japan, Jersey, Kiribati, North Korea, Laos, Latvia, Liechtenstein, Lithuania, Luxembourg, Macau, Macedonia, Malawi, Malta, Marshall Islands, Mauritius, Mexico, Micronesia Fed States, Moldova, Monaco, Mongolia, Montenegro, Montserrat, Namibia, Nepal, Netherlands, New Zealand, Nicaragua, Norfolk Island, Norway, Panama, Papua New Guinea, Paraguay, Peru, Pitcairn Islands, Poland, Portugal, Puerto Rico, Romania, Russia, Rwanda, Saint Barthelemy, Saint Helena Ascension, Saint Kitts & Nevis, Saint Lucia, Saint Martin & Tristan, Saint Pierre & Miquel, Saint Vincent & Grenadines, San Marino, Sao Tome & Principe, Serbia, Seychelles, Singapore, Sint Maarten, Slovakia, Slovenia, South Sudan, Spain, Sri Lanka, Suriname, Svalbard, Swaziland, Sweden, Switzerland, Taiwan, Thailand, Timor-Leste, Trinidad & Tobago, Turks & Caicos Is, Uganda, Ukraine, United Kingdom, Uruguay, Venezuela, Vietnam, Virgin Islands, Wallis & Futuna, Zambia, Zimbabwe (143 countries in total).
3. Third, in at least some of the societies that practice female (and hence also male) genital modification, there is, as alluded to previously, no “cultural obsession with feminine chastity, virginity, or women’s sexual fidelity [and] the role of the biological father is considered marginal and peripheral” [17] (p. 285).
4. Fourth, in some of the most highly patriarchal societies, *only* male genital modification is practiced, because the ritual is seen as a privilege to which boys and men alone are “entitled” (as in Orthodox Judaism) [55–57]. Thus, in at least some cases, patriarchy is associated with the *exclusion* of women and girls from genital modification, where this exclusion directly reflects their lower status [55].

From these four points alone, in combination with those raised in the previous sections, it should be clear that any overarching claim about a relationship between patriarchy and female genital modification should be treated with caution. This point can be illustrated with a comparison. Consider Egypt, Saudi Arabia, and Sudan: three large countries on the Red Sea with predominately Muslim populations. In all three countries, female virginity and marital fidelity are highly valued. All three practice male circumcision. Yet they offer marked contrasts in their female genital modification practices as well as their patterns of patriarchy.

Egypt has high rates of female genital cutting corresponding to WHO Type 1. But in Saudi Arabia, which has an overtly patriarchal, restrictive system of male guardianship of women that limits their freedom of movement and association, female genital modification is not customary among indigenous Arabs and is rare [120]. Meanwhile, Sudan has a very high prevalence of female genital modification (about 85%) and by far the dominant type is infibulation [121]. While all three countries might be judged to be dominated by male ascendency in political, economic, and social realms, Egypt and Sudan have many freedoms for women, while the most stringent control of women is that displayed in Saudi Arabia, which—as mentioned—has little native female genital cutting [122–124].

To summarize, it seems that neither the extent of male dominance within a society nor (asymmetrical) expectations of female sexual restraint allow one to assume a causal connection between those factors and the existence or prevalence of female genital modifications. Put another way, the fact that “FGM” is sometimes practiced within societies that are highly patriarchal does not entail that the practice, as a rule, is best *explained* by patriarchy or gender inequality, nor that it is primarily a means of upholding either.

### Patriarchy and genital modification: a cross-cultural comparison of preindustrial societies

Now we turn to our analysis of pre-industrial societies. Our reason for undertaking an illustrative discussion of pre-industrial societies is as follows: although female and male genital modifications began in pre-history, such that their “original” purposes and meanings are impossible to establish with any certainty, it is necessary, in any contemporary analysis, to account for the fact that economic development, industrialization, and Western colonialism have substantially reshaped social norms in many practicing societies [95].

For example, some scholars argue that binary, sex-based divisions of males and females into corresponding gendered social roles, with men positioned hierarchically over women in terms of status and power, was not a characteristic social arrangement in many pre-colonial African societies, where sex was not systematically associated with such distinctive and socially generalized roles, and power hierarchies operated, instead, primarily over age or seniority [95, 125]. To understand how male and female genital modifications are structurally related to each other, and to shed light on the range of cultural purposes they may serve, it seemed relevant to examine, not only present-day associations between genital modification and putatively patriarchal social arrangements, but also historical patterns prior to widespread European colonial “civilizing” and “developmental” projects [126].

We turned to Whyte’s [127, 128] cross-cultural comparative studies of the status of women in 93 pre-industrial societies. His cross-cultural method makes use of the Human Relations Area Files (HRAF) (https://hraf.yale.edu/), a data archive of classified cultural elements based on written ethnographic accounts of 400 well-described cultures from all parts of the world, offering examples of discrete cultural adaptations or ways of life. The unit of analysis is a single named ‘culture’ regardless of its relative size. Therefore, observations cannot be interpreted as percentages of humanity, but rather whether a given cultural practice is frequent or rare among the ways humans have developed cultural systems, and whether that feature of a society correlates with other variables of interest. The HRAF data have often been used for comparative cultural studies like Whyte’s, in which researchers draw samples based on their research questions. For example, Cody Ross and colleagues [129] used HRAF data to investigate possible origins and reasons for continuance of female genital modification (“FGMo” in their shorthand), finding that FGMo is slightly more likely to have developed in stratified societies, theorizing that it is a signaling ritual related to virginity and fidelity in the marriage market. However, they state that their findings must be “tempered by the finding that FGMo has arisen in many cultures that have no social stratification, and that forces operating orthogonally to stratification appear to play a more important role in the cross-cultural distribution of FGMo” (p. 173). Moreover, their analysis did not shed light on our more specific question about gender equality, potential sexual double standards, and female genital alteration customs.

Whyte’s sample is global and representative of the cultures of the world. While patriarchy in the political realm has been more or less global in a geographical and historical sense, it is not necessarily global (i.e., universal, or general) in its reach across domains within a society. Indeed, at least within the sample of preindustrial cultural groups studied by Whyte, patriarchy does not typically encompass all spheres of social life. Based on his coded data from available ethnographic evidence, Whyte discovered that gender inequalities in power, status, control, and agency are domain-specific and are not highly correlated across the many and diverse spheres of life (political, economic, legal, religious, sexual, familial). For example, although in any given society men may hold positions of political leadership or control primary religious rituals, it might be women who do healing rituals, make decisions about allocation of crops or the use of family resources, or control who their children marry. In other words, Whyte discovered that in pre-industrial societies there was no overall evidence of a highly generalized subordination of the status of women to men across the spheres and activities of social life.

Illustrative of the importance of comparative cross-cultural research to investigate the connection between customary female genital modifications and patriarchy is Whyte’s coded data on the following two variables, which we selected for inspection and illustration in this paper: variable #48 (titled “Existence of General Female Initiation Ceremonies”) and variable #21 (titled “Is there a double standard in regard to premarital sex?”). This illustration has a bearing on claims about the relationship between female genital modifications and male control of female sexuality.

The double standard on premarital sex—whereby boys and men are allowed to have premarital sexual relations (whether through active encouragement or more passive tolerance), while girls are punished or otherwise bear highly negative consequences for pre-marital sex—is an important marker of gender status, since it bears on freedom and autonomy. One might expect that a society that has a single standard for both sexes (whatever that standard might be) is more egalitarian than one that has a different standard for each (i.e., a “double standard”).

Whyte’s variable #48 on the presence or absence of female initiation ceremonies has the structure of a Guttman scale. His unanalyzed coded data from 93 pre-industrial societies [127], which was published and thus archived in the journal *Ethnology*, is presented in such a way that one can compare variations in premarital sexual standards (a double standard versus a uniform standard for males and females) across societies for which there are either “no initiations for females” (31 societies are coded that way in Whyte’s global sample) versus societies for which there are quite elaborate female initiation ceremonies (8 societies are coded that way in Whyte’s global sample). In those eight societies, the female initiations are elaborate in the sense that there is “personal dramatization” of the female initiate, an “organized social response” and what Whyte describes as an “affective social response (e.g., punishment or operations).” Contrary to what might be expected given the WHO’s generalizations in this area, we find no relationship between the presence of such female initiations and the existence of a sexual double standard.

Of course, none of these data are conclusive. The number of societies in Whyte’s sample with elaborate ritualized public ordeal-like initiations for females is small; and it is not entirely clear whether every one of those societies included a genital modification, although clearly some did. Nevertheless, it is eye-opening to discover in Whyte’s own analysis that a sexual double standard for males versus females with regard to premarital sex is not a universal (or even the most common) sexual norm among his global sample of pre-industrial societies. It is just as striking to discover, as we did by inspecting his archived coded data, that a single non-discriminating sexual standard for premarital sex prevails in seven of the eight societies with elaborate female initiation ceremonies (including those that involve female genital modifications).

Judging from Corrine Kratz’s [130] detailed ethnographic account of an elaborate female initiation ceremony with genital modifications among the Okiek of Western Kenya, many of those “cut” female adolescent initiates in Whyte’s sample may well have had boyfriends and premarital sexual partners who cheered them on and encouraged them to be stoical and courageous during their status-transforming and socially staged “rite of passage”. A similar dynamic has been reported more recently among the Jola of Casamance, Senegal by Liselott Dellenborg and Maria Frederika Malmström [54]. After noting that “Boys as well as girls are expected to accept the pain associated with circumcision [the purpose of which is] to help children achieve a good life by learning early on how to endure hardships” (p. 164), they write:

Chastity is not particularly highly valued in Jola society, and married women are permitted to take a lover (*asangor*) during the ritual, although this should be done with discretion and their husbands are rarely keen on it. Women explained the custom [as a] socially accepted way of meeting your ‘high-school lover’.… The resistance expressed by men is multifaceted and their criticism makes the women suspicious: are men opposed to the genital cutting itself or to the freedom women gain through their communal bonding in the initiation rite? (p. 170 and pp. 171–2).

Of course, such descriptions should not be generalized. Each culture that practices female—and thus also male—genital cutting must be understood on its own terms, taking into consideration local histories, social and economic arrangements, gender relations, and cultural narratives [50, 131]. Evidence of the sort mentioned above is simply a caution against rushing to judgment on the basis of received assumptions about the connection between patriarchy and customary female genital modifications.

## Conclusion

The patriarchy hypothesis is inconsistent with, and even contradicted by, much of what is currently known about female genital modification practices, both in terms of contemporary and pre-industrial societies. One possible, albeit partial explanation for the continued popularity of this hypothesis within the Western discourse is that it plays on dominant stereotypes about “backward” African cultures in need of rescue or reform from supposedly more enlightened nations (thereby justifying Western-led interventions to reshape African social, political, and economic systems under the “civilizing” banner of modern development) [126, 132–135]. Consistent with this possibility, there has been an overwhelming focus, within that discourse, on the subset of contemporary societies that are (a) highly patriarchal, (b) practice female genital modification (especially infibulation, the form most closely associated with female sexual control) while (c) ignoring the practice of male genital modification in the same societies [136], and (d) presenting this situation as typical of all societies that practice female genital modification.

Women and girls in many cultures face numerous inequities on account of their gender, ranging from unequal access to higher education or the marketplace, to greater rates of intimate partner violence and exclusion from certain forms of political power. However, where they have been incorporated into a gender-inclusive ritual [70] that involves genital modification of both sexes, this may constitute one of the *least* appropriate examples of a cultural practice that can monolithically be described as either reflecting “deep-rooted inequality between the sexes” or constituting “an extreme form of discrimination against women.” We suggest that efforts to discourage customary female genital modifications in societies where they remain common (without further disempowering affected women and girls by inadvertently weakening female-centered communal networks and bonding) will not progress if misleading generalizations continue to be used as a starting point for research or advocacy in this area [137]. We call on the WHO, journalists, and other analysts to avoid unsubstantiated generalizations about this female custom, and instead recognize the multiplicity of cultural meanings and contexts of both male and female genital alterations.

## Data Availability

The data presented in this paper are publicly accessible from the cited references.

## Footnotes

1 Almost all cultures that practice customary female genital modifications also practice customary male genital modifications (but not vice versa) as we discuss below. The most common of the male practices is known, in the English-speaking world, as male circumcision, although what this involves in concrete terms (e.g., how much tissue is removed) varies from group to group. The associated female practices are known in English by many labels including female circumcision, female genital cutting, female genital surgery, or female genital mutilation (“FGM”). Some international organizations and the media have adopted a single label—typically “FGM”—to cover all female-affecting procedures that are non-normative within Western culture, despite considerable physical and symbolic differences between variants. Following current scholarly usage, we employ the terms female genital modification or cutting. For in-depth discussion and justification of this terminology, see [1].

2 There are contemporary debates about the ethics of medically unnecessary genital cutting practices carried out in childhood, that is, when the cutting is relatively (i.e., compared to adults) or entirely (when performed on infants and newborns) involuntary. Those who oppose such cutting increasingly argue that it is the lack of consent, rather than a specific level of harm or the sex of the affected individual, that should drive the moral analysis of these practices, which include, in addition to female genital cutting, routine and religious male circumcision and “normalizing” surgeries on children with intersex traits or differences of sex development [92–94]. Although each of us has contributed to these ethical debates in other publications, and differ in our views and conclusions, for the purposes of the present article, our aim is not to advance a specific argument about when or under what conditions customary genital modifications may be morally permissible or impermissible. Rather, it is to evaluate a common theoretical explanation of *why* female genital cutting is performed in various societies, in terms of its social functions, symbolic meanings, and so on.

3 There are other, more theoretically sophisticated accounts of patriarchy, including in forthcoming work by Robin Dembroff [118]. However, the broad characterization we have given is consistent with longstanding, popular understandings of the sort that seem to be invoked by international campaigns opposing non-Western-associated forms of female genital modification.
